# An Intergeneric Hybrid Between Historically Isolated Temperate and Tropical Jays Following Recent Range Expansion

**DOI:** 10.1002/ece3.72148

**Published:** 2025-09-10

**Authors:** Brian R. Stokes, Timothy H. Keitt

**Affiliations:** ^1^ Department of Integrative Biology University of Texas at Austin Austin Texas USA

**Keywords:** anthropogenic hybridization, climate change, evolution, range shifts

## Abstract

Shifts in species' ranges are creating novel ecosystems and previously unobserved species interactions. Documenting and understanding these novel interactions between species is an emergent priority of global ecological importance. We report a wild hybridization resulting from recent range expansion: a hybrid between Green Jay ( *Cyanocorax yncas* ) and Blue Jay ( *Cyanocitta cristata* ), charismatic and historically allopatric species whose ranges newly overlap in Texas. Morphological and genetic evidence indicate the hybrid individual resulted from the pairing of a female Green Jay and a male Blue Jay. Hybridization between these species is remarkable across vertebrate species, as such events typically occur between recently diverged populations, whereas the most recent common ancestor to Blue and Green Jays is estimated to have lived at least 7 million years ago. We believe this hybridization event joins a growing list of increasingly unexpected outcomes of contemporaneous range expansions fueled by anthropogenic global change. As birds are keystone species in ecological webs and reservoirs for zoonotic diseases, the creation of unique genomic contexts resulting from climate‐driven hybridization is a phenomenon of both scientific and practical importance.

## Graphical abstract

We document the first known hybridization event between the Green Jay ( *Cyanocorax yncas* ) and Blue Jay ( *Cyanocitta cristata* ), two distantly related species whose ranges now overlap in Texas due to climate‐driven range expansions. Genetic and morphological evidence confirm this unprecedented hybridization, which represents a remarkable breach of prezygotic isolation between species separated by over 7 million years of evolutionary history. This study highlights the transformative impact of anthropogenic climate change on biodiversity and evolutionary processes.

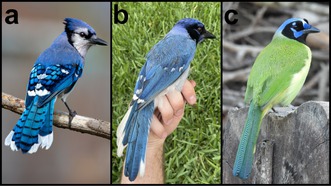

## 1 Introduction

Global heating is causing the expansion of the tropical climate zone (Staten et al. 2018) and tropical species are increasingly expanding their ranges away from the equator (Chen et al. 2011; McCarty 2001). Concurrently, changes in land use and land cover are driving range expansions of disturbance‐tolerant species affiliated with agricultural and suburban environments (Villarreal‐Barajas and Martorell 2009; With 2002). These dynamics may increase the frequency of interaction between tropical and temperate species. Such interactions can foster the development of novel ecological communities—often referred to as “no‐analog” communities—where species coexist in combinations that have not been previously observed (Williams and Jackson 2007). “Anthropogenic Hybridization” is one potential outcome of these no‐analog communities, supported by a growing body of evidence in both published literature and non‐peer reviewed reports (Larson et al. 2019; Parmesan 2006). Previous examples of anthropogenic hybridization have primarily focused on hybridization driven by human‐assisted species introductions and habitat modification via disturbance or altered fire regimes (Grabenstein and Taylor 2018; Ottenburghs 2021), with relatively few examples addressing hybridization driven indirectly by climate change (Chunco 2014). Our current knowledge of ecology and evolutionary mechanics provides a limited capacity to predict future outcomes under complex no‐analog conditions (Mouquet et al. 2015; Walther 2010) including the potential for novel hybridization between historically isolated species. Here, we report a previously undescribed case of hybridization in the wild between historically isolated tropical and temperate corvids facilitated by anthropogenic change.

To our knowledge, this is the first record of non‐captive hybridization between a Green Jay ( *Cyanocorax yncas* ) and a Blue Jay ( *Cyanocitta cristata* ). The Green Jay is a tropical species distributed from the northern Andes through Central America and Mexico into southern Texas. Green Jays are found in a limited region of Texas, where uniquely iridescent green and blue plumage makes them unmistakable and highly sought after by bird watchers. Thus, their range is well documented by citizen science datasets, such as eBird (Sullivan et al. 2009). Concordant with other species distributed at the tropical margin, Green Jays have remarkably expanded their range over the past two decades, shifting as much as 2 degrees of latitude over the span of a few generations (Sauer et al. 2022). Although experimental evidence of the role of climate change is lacking for most species range shifts, the region is warming rapidly, and we hypothesize that the lack of prolonged periods of freezing winter temperatures has released this species from its historical range limit in deep southern Texas (Osland et al. 2021; Rappole et al. 2011, 2007). The Blue Jay has similarly expanded its range to south and west Texas during a similar time period (Engels and Sexton 1994; Hutchinson and Scalise 2019). While climate change may have played a role in the range expansion of the Blue Jay, we note that this species has tracked the expansion of human settlement in other regions and is common in suburban gardens. Both species are commonly observed at artificial feeding stations, and thus we cannot rule out potentially interacting influences of climate and food subsidies on range dynamics.

A remarkable attribute of this observation is that unlike the majority of avian hybridization events documented in the wild, the Green and the Blue Jay are relatively distantly related and are not classified within the same genus. Phylogenetic analyses suggest that the ancestral lineages of these species diverged during the late Miocene, ~7.5 MYA (McCullough et al. 2022). Hybridization is relatively common among bird species (Grant and Grant 1992; McCarthy 2006), with approximately 16% of all bird species reported to hybridize in the wild (Ottenburghs et al. 2015), although this estimate is likely conservative due to underreporting. Most reported cases of hybridization rely on phenotypic observations, which can lead to substantial uncertainty when attempting to identify hybrids in the wild; thus, the use of genetic methodologies should be recognized as the gold standard for confirming hybrid identity and paternity (Ottenburghs 2021). Without genetic validation, even taxon experts can misidentify the paternity of hybrid specimens, as demonstrated by Alfieri et al. (2023).

The most phylogenetically divergent avian hybrid confirmed by genetic analysis is between the *Gallus gallus* (Domestic chicken) and *Numida meleagris* (Helmeted guineafowl), species which diverged ~51 to 65 MYA (Alfieri et al. 2024, 2023). An equally divergent hybrid of *N. meleagris* × *Pavo cristatus* (Indian peafowl) has also been described, but not confirmed through genetic analysis (Hanebrink et al. 1973). Non‐domestic species have also been observed to produce hybrids in captivity between highly divergent pairs, including a recorded cross between *Cardinalis cardinalis* (Northern Cardinal) × *Paroaria coronate* (Red‐crested cardinal) which diverged ~38 MYA (McCarthy 2006).

Instances of wild hybridization have been extensively recorded within the family Corvidae (McCarthy 2006; Ottenburghs et al. 2015). Corvidae includes 138 species with an estimated crown group divergence of ~10.8 MYA, according to phylogenetic analysis from McCullough et al. (2022). New World jays comprise a monophyletic grouping within Corvidae of ~38 species which diverged ~8.3 MYA (McCullough et al. 2022) and include the genera *Aphelocoma*, *Gymnorhinus*, *Cyanocitta*, *Calocitta*, *Cyanocorax*, and *Cyanolyca*. We identified reports of 26 intrageneric hybrids (crosses within the same genus) and 0 intergeneric hybrids (crosses between different genera) among Corvidae species outside the New World jay clade. Within the New World jays, we found 11 reports of intrageneric hybrids and two of intergeneric hybrids. Notably, there were no reports of crosses between New World jay species and other members of Corvidae. The two intergeneric hybridizations are *Aphelocoma californica* (California Scrub Jay) × *Cyanocitta stelleri* (Stellers Jay) and *Cyanocitta cristata* (Blue Jay) × *Aphelocoma coerulescens* (Florida Scrub‐jay) (Morgan and Morgan 1997). Both pairs diverged ~6.8 MYA (McCullough et al. 2022).

Previously, no *C. yncas* hybrids had been reported in the wild, but a hybrid *C. yncas* × *C. cristata* was produced in captivity in 1965 at the Zoological Park in Fort Worth, Texas (Pulich and Dellinger 1981). In the publication describing this hybrid the authors wrote “The possibility of the Green Jay and the Blue Jay occurring naturally together during the breeding season is remote, so that hybrids are not to be expected in the wild.” At the time of Pulich and Delliger's publication Green and Blue Jay breeding ranges would have been separated by about 200 km (Sauer et al. 2022). Here, we use genomic data to confirm the identity of the hybrid's parent species and to compare patterns of heterozygosity with other New World jay species. Additionally, we make use of citizen science and climate datasets to describe potential future range change and overlap between these species based on results from ecological niche models.

## 2 Materials and Methods

### 2.1 Observation

**FIGURE 1:**
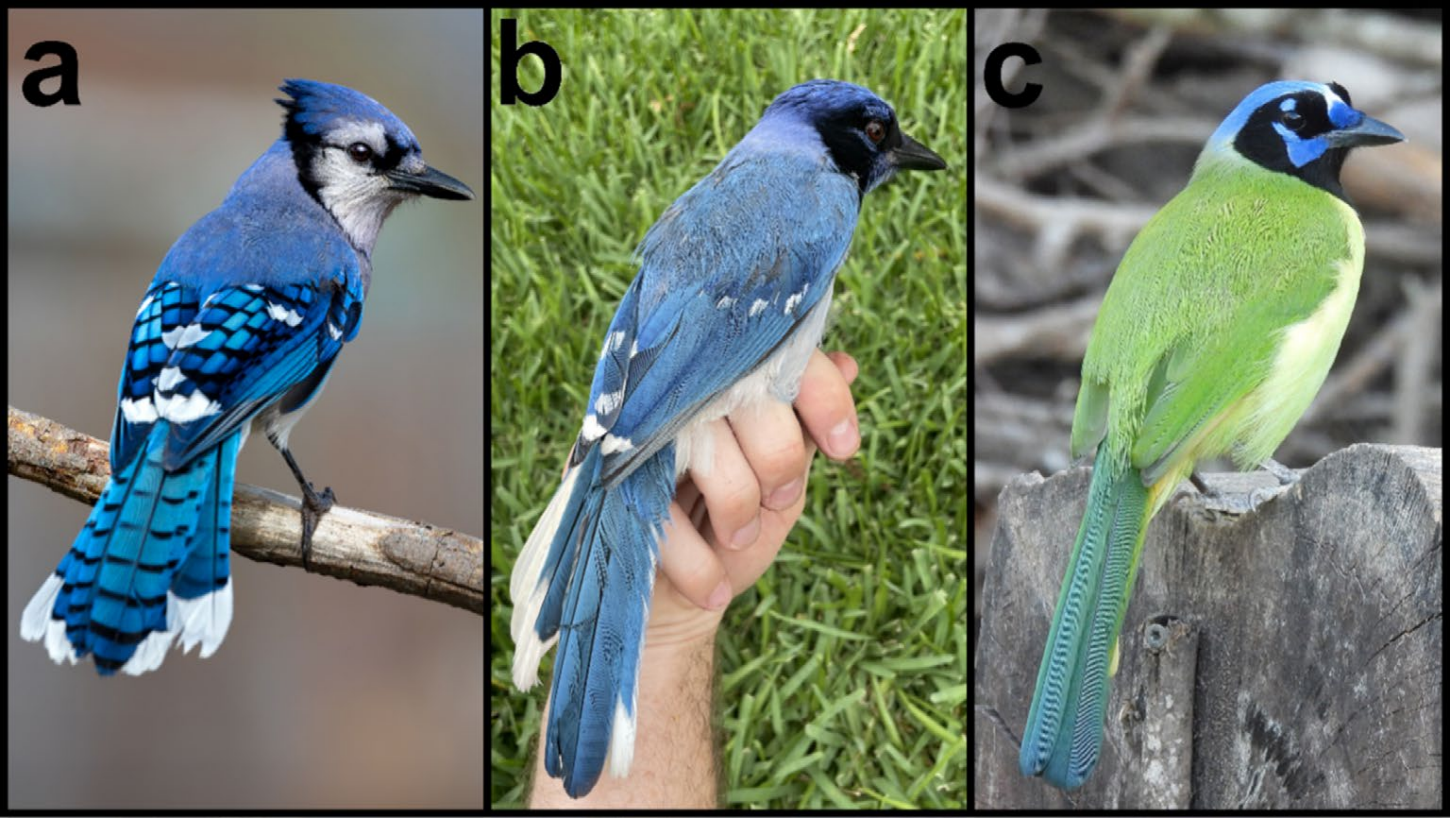
(a) Blue Jay by Travis Maher (ML578309451). Cornell Lab of Ornithology, Macaulay Library. (b) Hybrid Jay by Brian R. Stokes. (c) Green Jay by Dan O'Brien (ML390361871). Cornell Lab of Ornithology, Macaulay Library.

The hybrid was initially reported by a homeowner in a Facebook birding group “TEXBIRDS” in late May 2023. We observed and captured the hybrid using a mist net in early June 2023 at the homeowner's suburban property in Bexar County near San Antonio, Texas. Prior to capture, we observed the hybrid following a flock of Blue Jays closely across 2 days. The hybrid produced vocalizations similar to common Blue Jay calls, usually in response to Blue Jay vocalizations and activity. The hybrid also produced bill‐clicks and two‐tone low rattling vocalizations typical of Green Jays in Texas. Overall, the hybrid's plumage morphology was codominant intermediate, displaying distinct traits from both Blue Jay and Green Jay, rather than a blended phenotype (Figure 1). Specific feather tracts including the crown, nasal tufts, and longitudinal spot above the eye matched those of Green Jay, while the back and tail pattern matched those of Blue Jay. The hybrid lacked any yellow coloration, and its blue feathers resembled the dominant hues of a Blue Jay. The chin and upper throat of the hybrid were blue, which differs from both parent species. We judged the hybrid to be an after‐hatch year male based on molt characteristics, inner mandible coloration, and cloacal protuberance (Pyle et al. 2022).

### 2.2 Genomic Methods

We drew blood from the putative hybrid, attached a metal leg band, and released the individual at the location of capture. We extracted DNA from the blood sample using a Qiagen DNeasy Blood and Tissue Kit, following manufacturer protocol (Qiagen). We used an Illumina TruSeq Nano library preparation kit (Illumina) targeting a ~400‐bp insert size to generate short read data. We sequenced extracted DNA with a NovaSeq 6000 system with an S1 flow cell using single‐end 100‐bp sequencing chemistry targeting 110 million reads, approximately equivalent to 10× whole genome coverage. The raw read data are stored in the NCBI (BioSample accession: SAMN41474836). We cleaned the raw read data of adapter content using *fastp* v0.23.4 (Chen 2023). We aligned the reads to a Steller's Jay ( *Cyanocitta stelleri* ) reference genome (Genome Assembly bCyaSte1.0.p, GenBank assembly accession #GCA_026167965.1) (Benham et al. 2023) using *Bowtie2* v2.5.2 (Langmead and Salzberg 2012). Reads were aligned to the nuclear and mitochondrial reference scaffolds simultaneously (Zhang et al. 2016) to avoid misalignment of nuclear‐mitochondrial DNA segments.

We considered four candidate parent species for genomic comparison using BLAST+ (Camacho et al. 2009) analysis based on the proximity of plausible species range limits. These candidate species were Green Jay ( *Cyanocorax yncas* ), Blue Jay ( *Cyanocitta cristata* ), Steller's Jay ( *Cyanocitta stelleri* ), and Woodhouse's Scrub‐Jay ( *Aphelocoma woodhouseii* ). We also included Eurasian Magpie ( *Pica pica* ) as an additional outgroup for comparison of results. No genomic data for Woodhouse's Scrub‐Jay was available at the time of analysis, so we used a high‐quality California Scrub‐Jay ( *Aphelocoma californica* ) reference genome (GenBank assembly accession #GCA_028536675.1) (DeRaad et al. 2023) in its place.

To determine maternal ancestry, we created a representative mitochondrial sequence for each candidate species by using available sequencing data for BLAST analysis. We first collected mitochondrial scaffolds from reference genomes of California Scrub‐jay, Steller's Jay, Blue Jay (Genbank assembly accession #GCA_046129655.1; Rhie et al. 2021), and Eurasian Magpie (Genbank assembly accession #MT792356.1; Kryukov et al. 2020). We gathered raw whole genome sequencing data from four Green Jay samples (Bioproject accession: PRJNA1168985) that we had previously collected in Texas. We aligned the genomic data of each Green Jay to the Steller's Jay reference genome using *Bowtie2*, called variants using *BCFtools* v1.20 (Danecek et al. 2021), and used GATK v4.5 “FastaAlternateReferenceMaker” function (McKenna et al. 2010) to create a consensus Green Jay mitochondrial scaffold. We then added this scaffold and all other candidate mitochondrial scaffolds to a local BLAST database. We gathered autosomal sequences from each of the same sources but used a separate reference genome for Eurasian Magpie (Genbank assembly accession #GCA_025802055.1), as the previously used reference lacked autosomal data. We added all autosomal scaffolds to a single local BLAST database.

Next, we subset the putative hybrid reads which aligned to the reference mitochondrial scaffold. We generated a masked consensus sequence of the putative hybrid mtDNA using *BCFtools*. We determined masked positions by using the *Samtools* v1.20 “depth” function (Danecek et al. 2021) to identify bases below a sequencing score of 20 and/or below a mapping quality score of 20. Bases which fell below these thresholds were replaced with the IUPAC‐IUB nucleotide code “*N*,” which is treated as an unknown‐length gap by the BLAST+ algorithm. The resultant representative hybrid mtDNA sequence covered 41.81% of the reference mitochondrial scaffold, including partial coverage of the cytochrome *c* oxidase subunit 1 (*COI*), cytochrome *b* (*Cytb*), and NADH dehydrogenase 2 (*MT‐ND2*) genes. We used BLAST+ v2.14 to query the hybrid's mitochondrial consensus sequence against the local database of candidate mitochondrial scaffolds using default settings. The species with the highest sequence similarity was inferred to be the maternal parent. Additionally, we aligned mitochondrial scaffolds from the hybrid and candidate taxa with MAFFT v7.526 (Katoh 2002) to visualize sequence variation in a representative mtDNA region using *ggmsa* v1.12.0 (Zhou et al. 2022).

To determine autosomal ancestry, we used the GATK4 “HaplotypeCaller,” “GenotypeGVCFs,” and *Whatshap* v2.6‐0 (Martin et al. 2016) “phase” functions to perform haplotype assembly of the hybrid genome. The sequencing depth of the hybrid genome was insufficient to create long haplotype blocks when compared to reference scaffold length, leading to a high number of smaller haplotype phase blocks within each scaffold. To focus on informative regions, we retained phase blocks with 10 or more variant positions and a minimum length of 100 bp to use for blast analysis. We used the *BCFtools* “consensus” function to create a high‐quality consensus sequence for each haplotype of the putative hybrid within each phase block. We then queried both haplotypes of each phase block against the local candidate database with BLAST+ using default settings. To minimize bias arising from missing data across species, we restricted our BLAST analysis to regions homologous to the Steller's Jay scaffold #5 (GenBank: JANXIQ010000005.1). This scaffold covers a 55‐Mb region found on the 6th chromosome of each candidate species and was complete within each reference genome. After filtering by length and variant count, scaffold #5 contained 8827 phase blocks with an average of 12.51 variant sites (SD = 3.01) and an average length of 339.10 bp (SD = 117.58) per phase block. Under this approach, we expect a true F1 hybrid to exhibit roughly equal numbers of phase blocks showing the highest similarity to each of the two parental species since each haplotype should be inherited from a different species.

As a final step to determine parental ancestry of the hybrid, we used *trianglaR* v0.0.1 (Wiens et al. 2025) to calculate hybrid index and interclass heterozygosity of the hybrid, and to generate triangle plots. To minimize reference bias, we aligned and called variants against the *P. pica* genome for the hybrid, a single Blue Jay, and a single Green Jay. Hybrid index and interclass heterozygosity for the hybrid were calculated from ancestry informative markers (AIMs) defined by an allele frequency difference of *δ* = 1.0 between the Blue Jay and Green Jay, which restricted analyses to sites fixed for alternate alleles in each sample. This analysis was restricted to scaffold #23 (GenBank: JAOYNA010000001.1) which is one of the largest scaffolds in the reference genome and included a total of 1,526,230 AIMs. In this analysis, a true F1 Blue Jay × Green Jay cross is expected to have a hybrid index near 0.5 (equal ancestry from each parent) and an interclass heterozygosity close to 1.0 (heterozygous at nearly all AIMs). Sufficient deviations from these values could indicate either backcrossing or more complex ancestry. However, in our study, such deviations may also result from AIM selection based on limited parental data sources, as we defined AIMs based on single individual from each parental population. Standard applications of this method typically rely on larger parental samples to ensure robust identification of fixed differences between populations (Rosenberg et al. 2003).

Additionally, we calculated heterozygosity of the hybrid and each candidate genome within the Actin Beta (*ACTB*) gene. We extracted a homologous ~4.85‐kb region from each reference genome and the respective alternative reference haplotype. Only one haplotype was available for Eurasian Magpie, so we used a Western Jackdaw ( *Coloeus monedula* ) reference genome in its place (Genbank assembly accession #GCA_965178545.1). We selected the *ACTB* gene because it is highly conserved across each species and is expected to have undergone relatively slow evolution. We aligned the 2nd haplotype to the 1st haplotype of each species using bowtie2, called variants using *BCFtools*, and calculated heterozygosity by using the *BCFtools* “counts+” function. We calculated heterozygosity for the hybrid and each of the four Green Jay samples within the *ACTB* gene using similar methodology. Additionally, we created a “synthetic” hybrid *ACTB* region by aligning a single haplotype of the Green Jay sample with the highest coverage of the *ACTB* region with the first haplotype of the Blue Jay reference genome and calculated heterozygosity following the previously described methodology. This step was intended to compare an expected level of heterozygosity of a potential F1 hybrid with heterozygosity found in the natural hybrid.

We collected all genomic samples under appropriate state and federal permits and Institutional Animal Care and Use Committee (IACUC) protocols.

### 2.3 Ecological Niche Model

We used the eBird dataset (Sullivan et al. 2009) to evaluate the rarity of natural co‐occurrences between Blue Jays and Green Jays. We gathered all complete and non‐duplicate eBird checklists between January 1900 and May 31, 2023 (the approximate week the hybrid was first reported) containing observations of Blue Jay in Texas, Oklahoma, and Louisiana (*n* = 950,159) and observations of Green Jay in Texas and Mexico (*n* = 174,233). Checklists are the sampling unit of eBird, where checklist represents a single “birding event” or observational survey (Sullivan et al. 2009). We performed additional filtering to remove vagrants from the dataset (e.g., a vagrant Green Jay recorded in urban Houston). We cross‐referenced checklists and sampling localities to identify the sampling locations where both Green Jay and Blue Jay were observed in the same checklist. We then prepared data for MaxEnt modeling by excluding species observations from localities that did not meet either of two criteria: (1) the species was reported in at least two separate years between 2019 and May 31, 2023 or (2) the species was detected on at least 50% of checklists submitted at the same locality across the full eBird dataset. These filters aimed to limit the impact of vagrant occurrences without introducing strong bias toward localities with exceptionally high sampling effort.

We downloaded a current and a future projected climate normals dataset which included a total of 33 Bioclimatic variables at a spatial resolution of 1 km (Mahony et al. 2022). The “current” dataset includes the time period of 1991–2020, which represents the approximate climate in which both species have reached their current range limits. The future projected dataset is produced as an ensemble mean of 8 CMIP6 AOGCMs for the SSP2‐4.5 climate pathway, projected to the time period of 2041–2060. The SSP2‐4.5 pathway represents a future emissions scenario with minimal change from current global output trends (Riahi et al. 2017) which would result in a projected 2.03°C increase in mean annual temperature at the location where the hybrid was captured. We clipped all environmental layers to fit a region near the range boundaries of both species in central Texas (20°–35° N, 93°–105° W). We also removed the MAR (mean annual solar radiation) layer from the dataset because grid cells within Mexico were missing data.

We used MaxEnt v3.4.3 in *Dismo* v1.3‐14 (Hijmans et al. 2010) to quantify the correlation of Blue and Green Jay observations to current climate normals and project potential suitable climatic habitat based on these correlations. Additionally, we removed all Blue Jay observations prior to 2018 due to vector length constraints in R, which resulted in 76,895 observations after filtering. We used *GeoThinneR* v2.0.0 (Mestre‐Tomás 2025) to retain a single observation per 10‐km grid cell. To generate an absence dataset for each species, we identified all localities with at least one checklist submitted per year from 2019 to 2023 and selected the localities where the species had not been recorded during that period. We projected the results of the MaxEnt model for each species to the current climate and future climate datasets and converted model output rasters to polygons where values above each model's maximum sensitivity plus specificity threshold were converted to 1's and values below the threshold were converted to 0's.

## 3 Results

Sequencing resulted in 93,925,760 single‐end reads for the hybrid, 93.11% of which mapped to the *C. stelleri* reference genome. This produced an average coverage of ~7.52× across the genome, with over 99.48% of the genome sampled by at least one read.

**FIGURE 2:**
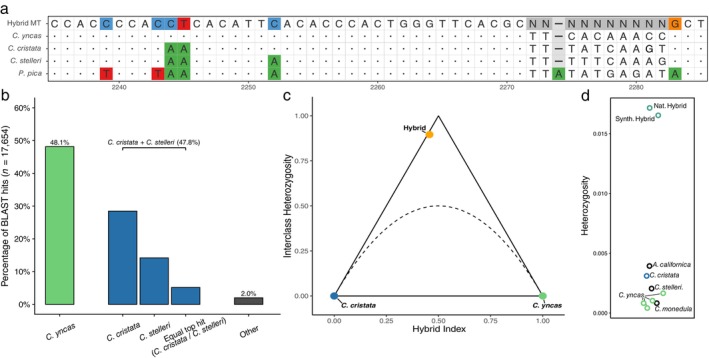
(a) Visualization of a representative 16S rRNA region from the MAFFT alignment. The hybrid mitochondrial sequence (“Hybrid MT,” top) is shown explicitly. For the other taxa, bases matching the hybrid are rendered as dots; non‐matching bases are shown as letters. Masked positions are “N,” and indels are shown as “–”. Colors denote base identity at variant positions. (b) Distribution of top BLASTN hits for 17,654 phase blocks from scaffold #5 (≥ 97% identity). Bars indicate matches to *C. yncas* (green), *C. cristata* (blue), *C. stelleri* (blue), and equal top hit ( *C. cristata* / *C. stelleri* )—phase blocks with equal bit score between the two species (blue), and other (gray). Other represents the additional candidate species and any combination of score ties aside from *C. cristata* / *C. stelleri* . Percentages are relative to all blocks; the bracket indicates the cumulative percentage for *C. cristata* + *C. stelleri* (47.8%). (c) Triangle plot of hybrid index values. Points represent *C. yncas* (green), *C. cristata* (blue), and the hybrid (orange). Placement of the hybrid near a 1.0 interclass homozygosity and 0.50 hybrid index strongly supports the hybrid to be a F1 hybrid between *C. yncas* and *C. cristata* . (d) Per‐site heterozygosity at the nuclear *ACTB* gene. Points show natural and “synthetic” hybrids (teal), *C. cristata* (blue), four *C. yncas* samples (green), and other candidate species (black). Points are horizontally jittered to reduce overlap; the x‐position has no meaning.

BLAST results for the mitochondrial reads of the hybrid matched most closely to the consensus mitochondrial scaffold of Green Jays in Texas (average 97.9% identity) followed second by Steller's Jay (97.4% identity). BLAST results for the autosomal phase blocks (≥ 97% identity; *n* = 17,654) indicate approximately even identity between *Cyanocitta* and *Cyanocorax* lineages (Figure 2b). 49.8% of phase blocks had top hits to *Cyanocitta* (Blue Jay, Steller's Jay, or a tie between the two species) and 48.1% to Green Jay; the remaining phase blocks matched other taxa or ties between other taxa. Reads from the hybrid genome were aligned to the Steller's Jay reference genome, so some reference bias toward Steller's Jay is expected, but Blue Jay still accounted for 28.5% of all top hits. Analysis from *triangulaR* calculated the hybrid to have a hybrid index of 0.46 and interclass heterozygosity of 0.89 (Figure 2c). Collectively, autosomal and mitochondrial results strongly support the assertion that the putative hybrid is an F1 cross between a female Green Jay and a male Blue Jay.

Heterozygosity within the *ACTB* gene was low compared to some genome‐wide estimates found in other avian studies (Toews et al. 2022). Despite this, the natural hybrid and “synthetic” Blue Jay × Green Jay hybrids contained high levels of heterozygosity compared to other corvid species (Figure 2d). The mean heterozygosity estimate for all other samples was 0.0017. The synthetic hybrid had a heterozygosity value of 0.0165, and the natural hybrid had a heterozygosity value of 0.0171.

**FIGURE 3:**
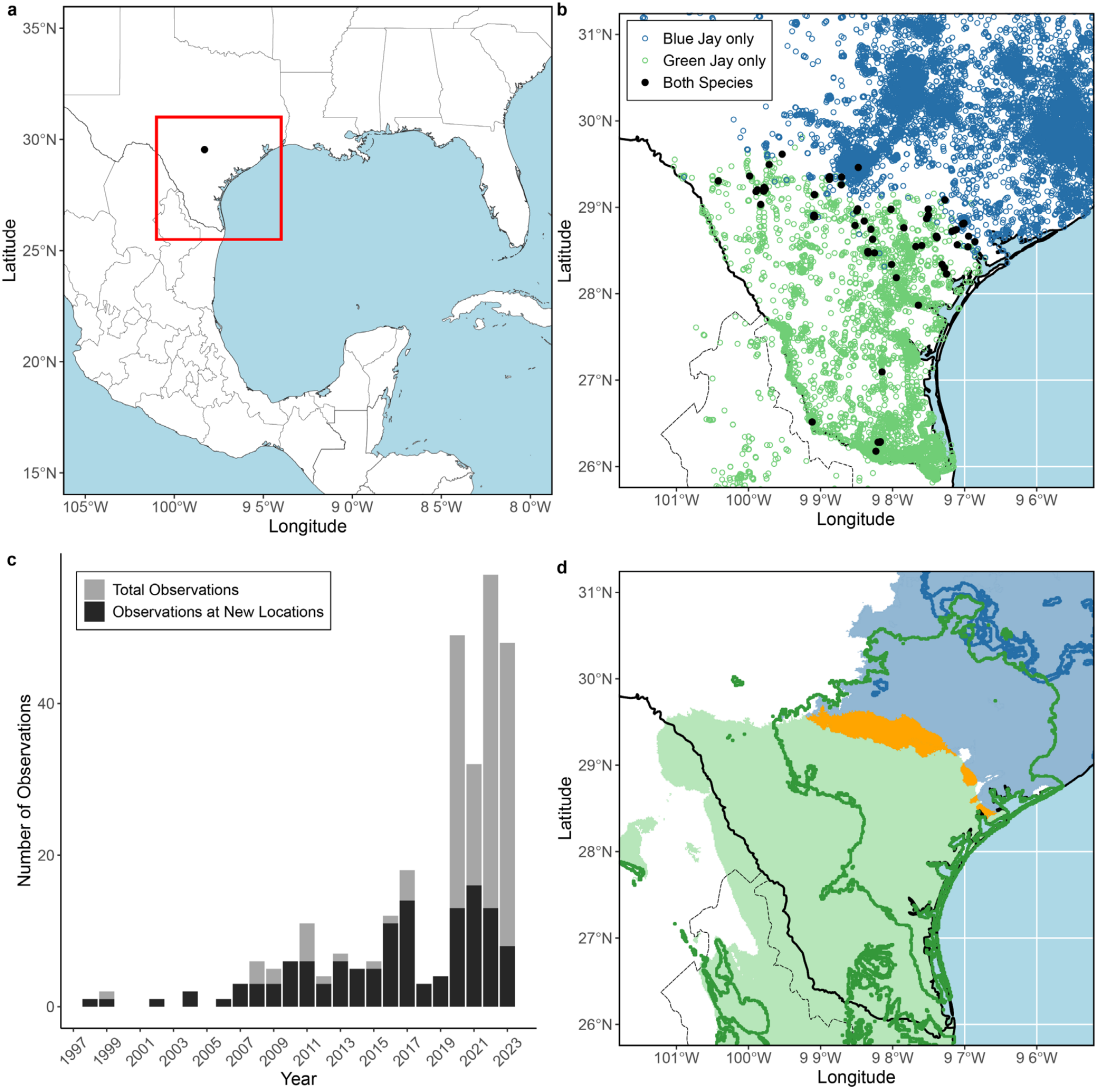
(a) Region of study. Red square delineates extent of maps for panels b and d. (b) Green Jay and Blue Jay occurrences, black points indicate localities of recorded co‐occurrence. (c) eBird recordings of Green and Blue Jay co‐occurrences per year. Gray bars are the raw count of all checklists each year which record a co‐occurrence. (d) Green and Blue Jay climate niche distributions based on MaxEnt using climate norms of 1991–2020 and climate norms for ssp245 emission projection for 2041–2060. Light blue and green areas represent current predicted climatic niches of Blue and Green Jay respectively. Orange area represents current predicted climatic niche overlap between both species. Blue and green lines represent boundary of future projected distributions.

Within the eBird dataset, we found 284 checklists at 79 unique sampling locations where both Green Jay and Blue Jay were observed in the same checklist (Figure 3b). Co‐observations generally increased from 2000 to 2023, although this trend is likely influenced in part by increasing participation and observational effort within the eBird dataset during the time period (Figure 3c). Additional observations of co‐occurrence have been reported between our data cutoff date (May 31, 2023) and the production of this report. An eBird checklist recorded during April, 2024 included both species and noted that the observed Blue Jay appeared as “[an] accepted member of [the Green] jay flock near feeder” (eBird checklist S170365878).

Finally, the MaxEnt models indicated that regions within the current Green Jay range show a strong correlation with lower levels of freezing weather and snow. When projected to the current climate dataset, the MaxEnt model produced a region of overlap between the Blue and Green Jay which covered an area of 5197.70 km^2^ with a centroid point at (29.39878, −98.13496). When projected using the future climate dataset, the region of overlap was comprised of a series of small patches covering a total area of 482.68 km^2^ with a centroid point at (30.46149, −96.35703) (Figure 3d), which is equivalent to an ~119 km northward shift.

## 4 Discussion

Expansion is a major mechanism leading to hybridization. Given the accelerating pace of global change, we expect that novel hybrids will be an important component of future ecosystems. Our observation of a novel hybrid with parents of distant ancestry highlights the increasingly surprising nature of rapidly changing ecosystems. We believe this individual to be the first described contemporary hybridization event between two socially complex vertebrate species driven by anthropogenic change.

We are not aware of additional sightings of Blue × Green Jay hybrids, which may reflect limited birding activity in the habitats most likely to contain potential hybrids. Based on morphology, we inferred the hybrid to be at least a second‐year individual, which indicates it had survived a full year without being reported to any public database. Between late summer of 2023 and June 2025, the hybrid was not seen by the original observer or reported to eBird, iNaturalist, online forums, or social media groups. It was resighted at the original site of capture on June 9, 2025, confirming its persistence. The majority of the region of potential sympatry is a sparsely populated area when compared to the suburban environment the hybrid was first reported in. Public land covers < 1% of this region, and observational effort as measured through eBird participation is relatively low. We argue that broad plumage similarity to locally common Blue Jay and limited public access to suitable habitat may reduce the likelihood of detection for additional potential hybrids.

Regardless of whether this observation reflects an isolated occurrence or is indicative of broader patterns that may emerge in the future, it joins a growing list of no‐analog interactions resultant of anthropogenic change (Williams and Jackson 2007). As species continue to shift their ranges in response to climate change, habitat shifts, and other ecological pressures, encounters among historically allopatric taxa may become increasingly common. Anticipating the nature and consequences of these novel interactions represents a central challenge for ecologists in the coming decades. Ultimately, our ability to forecast such outcomes will remain constrained by methodological limits and gaps in underlying mechanistic theory (Beckage et al. 2011; Mouquet et al. 2015). Our report of a novel species interaction further serves to highlight the urgency of documenting these emergent dynamics, which may foreshadow ecological shifts as climatic and broader anthropogenic changes continue to reshape biotic communities.

## Author Contributions

**Brian R. Stokes:** conceptualization (lead), data curation (equal), formal analysis (equal), funding acquisition (supporting), methodology (lead), visualization (lead), writing – original draft (lead), writing – review and editing (equal). **Timothy H. Keitt:** conceptualization (supporting), data curation (equal), funding acquisition (lead), visualization (supporting), writing – original draft (supporting), writing – review and editing (equal).

## Conflicts of Interest

The authors declare no conflicts of interest.

## Data Availability

All sequencing data utilized are publicly available at NCBI. Genomic and eBird data sources and code to produce results and figures are provided at https://github.com/brianstokesUT/Hybrid‐Jay.
